# How Incivility and Academic Stress Influence Psychological Health among College Students: The Moderating Role of Gratitude

**DOI:** 10.3390/ijerph17093237

**Published:** 2020-05-06

**Authors:** Naizhu Huang, Shaoping Qiu, Amin Alizadeh, Hongchao Wu

**Affiliations:** 1 Higher Education Institute, Xiangnan University, Chenzhou 423000, China; huangnzh@163.com; 2 The Department of Engineering Technology & Industrial Distribution, Texas A&M University, College Station, TX 77843, USA; 3 The Department of Educational Administration & Human Resource Development, Texas A&M University, College Station, TX 77843, USA; ameen59@tamu.edu; 4 School of Education, South China Normal University, Guangzhou 510631, China; wuhc@scnu.edu.cn

**Keywords:** incivility, academic stress, psychological health, gratitude, college students, China

## Abstract

Many students suffer from academic stress and uncivil behaviors at colleges and there is a need to identify to what extent these negative phenomena might impact students’ mental health. The main purpose of this study is to examine the relationships between incivility, academic stress, and psychological health, as well as investigate the moderating role of gratitude. The study design of this research is cross-sectional. The final sample consisted of 895 university students in China; The Statistical Package for the Social Sciences (SPSS) version 22 was utilized to conduct statistical analysis. Sample t-tests were used to examine whether there were gender differences in terms of four continuous variables: incivility, stress, gratitude, and psychological wellbeing. We also used multiple hierarchical linear regression analysis to test the relationships between the aforementioned four variables and the moderating effect of gratitude. The results of our study indicate that academic stress and incivility are positively associated with psychological distress, and gratitude moderates the relationship between incivility and psychological distress. However, no significant moderating effect of gratitude was found in the relationship between academic stress and psychological distress.

## 1. Introduction

The problems of psychological distress are prevalent across the globe and have been the major causes of disproportionately higher rates of disability and mortality [1]. As a set of painful mental and physical symptoms, psychological distress includes anxiety, confused emotions, hallucinations, and depression [2]. Statistics on psychological distress are alarming; mental illness accounts for 30% of non-fatal disease burdens worldwide and 10% of overall disease burdens, including death and disability [3]. The proportion of the global population with depression in 2015 was estimated to be 4.4% [4]. The problems of psychological distress are especially prominent among college students, as they are particularly more vulnerable to psychological disorders [5]. For example, in China alone, about 20% of college students suffer from various forms of mental problems, such as depression, anxiety, and stress [6]. Therefore, psychological distress of college students has become an issue of utmost concern worldwide [7].

In parallel, the topic of psychological distress has drawn much attention among researchers and practitioners worldwide. Research has demonstrated that stress perceived as a personal threat and loss of control contribute to problems of psychological distress [8]. Psychological stress derives from a threat to losing control of resources and lack of gained resources, according to the Conservation of Resources Theory [9]. Therefore, individuals who perceive themselves as having a large amount of pressure are more prone to develop psychological discomfort and negative emotions [10]. This argument is corroborated by mounting empirical evidence. For example, a meta-analysis study conducted by LePine et al. [11] demonstrated that stress is positively related to psychological strain, such as anxiety, depersonalization, depression, and emotional exhaustion. Recently, Wu et al. [12] identified that both challenge and hindrance stressors have detrimental effects on psychological distress among schoolteachers in Chinese schools, as they see these stresses as a threat or have little control over them. As college students transition into young adulthood, they are more likely to suffer from many stresses because during this process they need to strike a balance between competing demands related to academics, social relationships, and personal needs [13,14]. Most of the time, academic stresses such as those imposed by academic assignments at schools pose a huge threat for college students, as they view having little control over the situation.

Like academic stress, uncivil behaviors or comments can arguably be considered as one type of stress that individuals might socially encounter on a regular basis [15]. Incivility refers to rude speech or behavior, impoliteness, bad manners, and inappropriateness [16]. It is a form of discourteous communication that violates the norms of mutual respect. In the context of college, incivility is instigated by classmates and other college students or faculty members. Even though some of these uncivil behaviors or language might appear vague as far as intent to harm, the recipients most likely feel threatened depending on their personality traits, state, and social support. Lane and McCourt [17] contended that incivility may contribute to increased health problems. A growing body of empirical research provides strong support for this argument. For instance, treating customer incivility as a specific minor daily stress, Arnold and Walsh [18] found a positive association between customer incivility and employees’ psychological wellbeing. Studying daily email communications, Park et al. [19] revealed that employees reported higher levels of affective, physical distress at the end of the workday after experiencing cyber incivility, resulting in higher distress even the next morning. A recent integrative review conducted by Rose et al. [20] investigated student-to-student incivility. They disclosed that students who encounter incivility tend to experience a variety of physical, emotional, and psychological stress.

While both academic stress and incivility pose potential external threats to people who subsequently might experience psychological harm, gratitude could be inversely related to indicators of psychological distress, such as stress and depression [21,22]. Gratitude is defined as an affective human characteristic directed towards other people that results from a proactive acknowledgement that one has obtained a positive outcome and others have made certain sacrifices for this positive outcome [23]. Emmons [24] suggested that a grateful attitude towards life tends to provide protection when people are facing hardship and turmoil and allows for the development of a more powerful resilience to deal with adversity and crisis. The expression of appreciation has the potential power to transform stress into opportunity [25]. Individuals with high gratitude to others are more likely to appreciate daily events in life, which in turn could facilitate adaptive coping to curb stress [26,27]. In other words, gratitude can play a buffering role in the relationship between stress and psychological distress [28].

**Figure 1:**
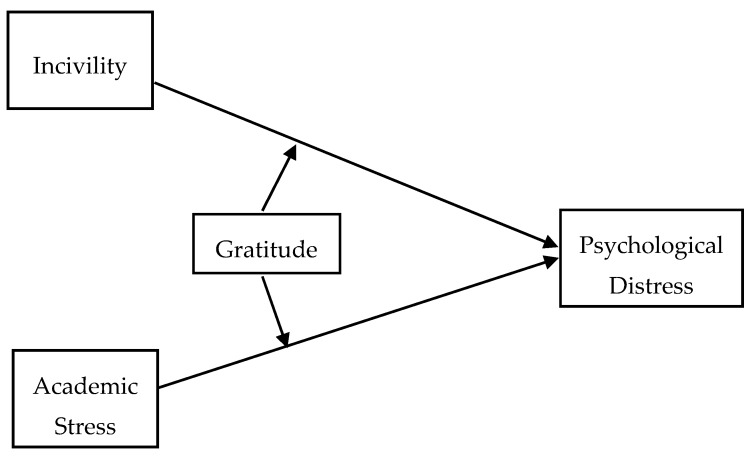
Conceptual model.

In this study, we especially focus on Chinese college students. The purpose of this study is to examine relationships between incivility, academic stress, and psychological health, as well as investigate the moderating role of gratitude. To this end, we proposed the following hypotheses based on the reasoning and literature above. Hypothesis 1: There is a positive association between incivility and psychological distress. Hypothesis 2: There is a positive relationship between academic stress and psychological distress. Hypothesis 3: Gratitude moderates the relationship between incivility and psychological distress. Hypothesis 4: Gratitude moderates the relationship between academic stress and psychological distress. Both positive relationships of incivility and academic stress with psychological distress would be weaker for those who express more gratitude to others in life. The conceptual model is provided in Figure 1.

## 2. Materials and Methods

### 2.1. Participants and Procedure

The study design of this research was cross-sectional. Data were obtained from students in November 2019 at a large university in South China. Since the survey was administered in Chinese, and we used instruments originally developed in Western countries, we conducted a back-translation to guarantee that the meaning of the instrument items was accurate and culturally appropriate. Prior to conducting the survey, a pilot study was carried out using 40 students to assess clarity, length, comprehensiveness, and completion time of the measures. The survey instrument was distributed via the internal email listserv of the university to 19,532 students who came from all provinces of China. A total of 1425 students participated in this study and returned their responses. After excluding incomplete questionnaires, we obtained 895 valid, usable data cases with a response rate of 4.58%. Therefore, the final sample consisted of 895 university students.

Among participants, 695 (77.7%) were female students. The average age was 19.14 years old. In total, 392 students (43.8%) were freshman while 352 (39.3%) were sophomores. In addition, 357 participants (39.9%) reported that their monthly family income was between 3000 to 5000 RMB Yuan, while only 37 respondents reported their family earned 11,000 or above RMB Yuan per month. Most students reported that their father (40.6%) and mother (40.1%) only graduated from middle school. A vast majority of students (493, 55.1%) originally came from the province where the university is located. However, the remaining participants were roughly evenly distributed among other provinces of China except for three autonomous regions.

### 2.2. Measures

All measures employed were developed and validated and used in previous studies. In addition to ensuring participating students’ anonymity and confidentiality, we used a few negatively worded items in the survey to reduce common method variance [29]. Incivility was assessed using a seven-item scale developed by Cortina et al. [30]. Students were asked to rate the frequency they have been in a situation where any of their classmates exhibited uncivil behavior to them. The rating scale of 1 (never) to 5 (very frequent) was used. An example item was: “How often did my classmates make demeaning or derogatory remarks about me in the last year?”. The reliability of this scale was acceptable with an alpha of 0.86.

Stress was measured using a 10-item scale developed by LePine et al. [31]. Students were required to rate the extent to which they experienced academic stress on a scale of 1 (no stress) to 5 (a great deal of stress). Two example items were: “The amount of hassles I need to go through to get projects or assignments done” and “The difficulty of the work required in my classes” Cronbach’s alpha was acceptable at 0.89.

Gratitude was evaluated using the Gratitude Questionnaire-Six Item Form (GQ-6) developed by McCullough, Emmons, and Tsang [32]. Participants rated the extent to which they agreed with statements on a scale ranging from 1 (strongly disagree) to 5 (strongly agree). One example item was: “I have so much in life to be thankful for” The measure had an adequate internal consistency (α = 0.83).

Psychological distress was measured using K6 screening scale developed by Kessler et al. [33]. Students rated on a scale of 1 (never) to 5 (very often) how often they felt psychologically distressed, such as hopeless. The reliability of this scale was 0.88.

Control variables included age, gender, and family income. These variables were found to predict psychological distress in previous research [33,34,35]. For gender, we coded male as “1” and female as “2”. In terms of age, we coded age 18 as “1”, age 19 as “2”, age 20 as “3”, age 21 as “4”, age 22 as “5”, and age 23 or above as “6”. With respect to family income, we coded family monthly income below 3000 RMB Yuan as “1”, 3000–5000 as “2”, 5000–7000 as “3”, 7000–9000 as “4”, and above 9000 as “5”.

## 3. Methods

SPSS version 22 was utilized to conduct statistical analysis. Mplus was used to test convergent and discriminant validities of the main variables. Independent sample t-tests were used to examine whether there were gender differences in terms of the four continuous variables: incivility, stress, gratitude, and psychological wellbeing. We also used multiple hierarchical linear regression analysis to test relationships between the aforementioned four variables and the moderating effect of gratitude, controlling for age, gender, grade, and household income. The *p*-values ≤0.05 were considered throughout as statistically significant. We used Harman’s single factor test to check whether there was common method variance in the data. Results revealed that one single factor only explained 26.86% of the variance, much lower than 50%, indicating no major issues with common method variance. Means, standard deviations, reliability, and intercorrelations between study variables are shown in Table 1.

As can be seen from Table 1, gender is associated with gratitude (r = 0.15, *p* ˂ 0.01). However, it has no significant relationship with incivility, stress, and psychological wellbeing. Psychological distress is significantly related to incivility (r = 0.37, *p* ˂ 0.01), stress (r = 0.52, *p* ˂ 0.01), and gratitude (r = −0.20, *p* ˂ 0.01), whereas correlation between stress and gratitude is not statistically significant.

Confirmatory factor analysis (CFA) was performed to test convergent and discriminant validities of main variables (i.e., incivility, academic stress, gratitude, and psychological distress). For this purpose, we compared four measurement models. In the three-factor model, we combined incivility and academic stress because they were two independent variables. In the two-factor model, incivility, academic stress, and gratitude were put together as one variable. The fit indices of all four models are shown in Table 2. As can be seen from this table, the four-factor model provided a good fit with the data and was much better than any other models (χ^2^ = 502.17, *df* = 203, RMSEA = 0.04, CFI = 0.96, TLI = 0.96, SRMR = 0.04). Thus, the discriminant validity was established. In addition, the factor loadings in the four-factor model were all greater than 0.50 and all values of average variance extracted (AVE) for the four variables were also greater than 0.50. Therefore, convergent validities were achieved for all four variables.

## 4. Results

Results from independent sample t-tests revealed that there were no statistically significant differences between male and female students in terms of incivility, stress, and psychological wellbeing. However, discrepancies between male and female students were significant, with female participants reporting more gratitude towards others (t = −4.43, *p* ˂ 0.001).

Hierarchical multiple regression analysis was performed to examine the relationships between incivility, stress, gratitude, and psychological wellbeing, as well as the moderating effect of gratitude. We mean-centered the values of incivility, stress, and gratitude [36]. Results are presented in Table 3. As seen from this table, there was a significant main effect of incivility in both Model 2 (β = 0.22, *p* ˂ 0.01) and Model 3 (β = 0.22, *p* ˂ 0.01), indicating that students feel more psychologically unhealthy under high-level incivility from their classmates. We also found that a significant effect of stress in both Model 2 (β = 0.46, *p* ˂ 0.01) and Model 3 (β = 0.46, *p* ˂ 0.01) revealed that students experience more psychological distress issues under high academic stress. In addition, the effect of gratitude on psychological distress was also negatively significant in both Model 2 (β = −0.18, *p* ˂ 0.01) and Model 3 (β = −0.17, *p* ˂ 0.01), indicating that college students who express more appreciation to others suffer less from psychological distress problems. Most importantly, the interaction term between incivility and gratitude was significant in Model 3 (β = 0.07, *p* ˂ 0.05). However, there was no significant moderating effect of gratitude on the relationship between incivility and psychological wellbeing.

**Figure 2:**
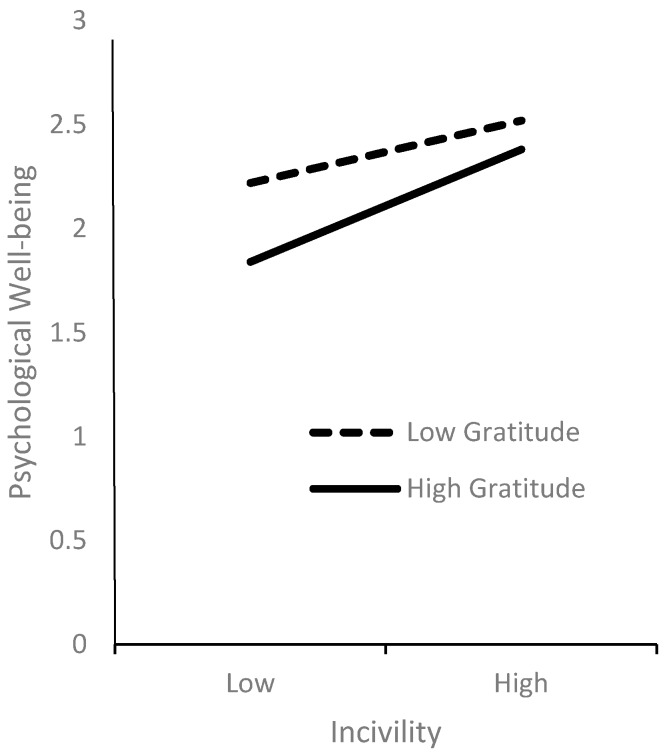
Moderation effect between incivility and gratitude on psychological wellbeing.

Following the guidelines of Aiken and West [36], we plotted the regression of psychological distress on incivility to assess the moderation effect at two values of gratitude (mean +1 standard deviation and mean −1 standard deviation) (Figure 2). As illustrated in this figure, the higher the level of gratitude, the stronger the relationship between incivility and psychological wellbeing. When incivility is low, students with a low level of gratitude experience more severe psychological distress problems. However, as the level of incivility becomes higher, psychological distress issues become less different across these two gratitude groups.

## 5. Discussion

Using a sample of 895 students recruited from a university in South China, we investigated the relationships between incivility, academic stress, gratitude, and psychological distress. In addition, we examined underlying mechanisms through which incivility and academic stress affect students’ psychological distress. That is, we tested how incivility and academic stress interact with gratitude to predict students’ psychological distress. Moreover, we compared differences between male and female students with respect to incivility, academic stress, gratitude, and psychological distress.

Results of this study illustrate that incivility is positively associated with psychological distress. Our findings are consistent with prior studies [18,19,37]. Academic stress was also confirmed as related to psychological distress. This conclusion is also in line with previous research that examined the aforementioned relationship [11,12]. It was also identified that gratitude moderates the relationship between incivility and psychological distress. However, no significant moderating effect of gratitude was found in the relationship between academic stress and psychological distress, which is contrary to our expectations.

In low incivility environments, grateful students are less likely to suffer from psychological distress than those with low levels of gratitude. It is sensible because under normal conditions, grateful individuals tend to hold a positive attitude towards life, take more pleasure from benefits in life, and feel happier [38]. As uncivil behaviors or comments become relatively more frequent, grateful students can still keep psychologically healthier. However, as incivility frequency increases, the psychological distress college students experience increases more rapidly for grateful students. A possible reason would be that grateful individuals take uncivil communications more seriously and attempt to resolve conflicts to maintain high-quality interpersonal relationships with others [39]. Most probably they attribute sources of these uncivil behaviors and comments to themselves and ruminate on how they could change their thoughts, thus aggravating their psychological problems [40].

In this study, we did not find any gender differences on perceived incivility, perceived level of stress, and reported psychological health. The finding of no gender disparity on perceived stress and psychological distress contradicts the study results of Moksnes and Lazarewicz [28]. In their research on Norwegian adolescents from 13 to 18 years old, they found that boys scored lower than girls on stress and symptoms of depression and anxiety. These inconsistencies may be attributed to the difference of age range. Another plausible reason could be the emphasis Chinese universities place equally on both male and female students. In our study, the sample participants were Chinese college students who were developing or built their own resilience and coping strategies. In Chinese universities, there are counselors at both university and department levels dedicated to helping students with personal, emotional, and psychological concerns. When facing stress and adversity, both male and female students are likely to be equipped with the same skills to handle negative situations encountered and to perceive the stress as less severe. However, female college students, compared to male students, were found to be more likely to express gratitude towards others. This finding corresponds with previous studies showing that women tend to report higher levels of gratitude than men [41,42,43]. As reasoned by Watkins et al. [44], men generally associate gratitude with weakness in personality. Therefore, men tend to avoid expressions of gratitude to protect their masculinity and maintain their social status.

This study makes both theoretical and practical contributions. First, research examining the association between incivility, academic stress, and psychological distress in the Chinese context is scarce. Using Chinese college students as a study sample is even more scant. This study adds to the current literature by enhancing our understanding of whether incivility and academic stress influence students’ psychological distress in a Chinese university setting. Additionally, given that little is known about how incivility and stress affect psychological health, testing the moderating effects of gratitude helps us gain an understanding of boundary conditions under which such an association might occur. Therefore, this study might fill a theoretical gap in the literature. Third, findings of this study could provide insight and timely advice to Chinese university students on how to keep psychologically healthy. Especially during this difficult time of novel coronavirus outbreak, students are learning online from home. The study of gratitude and stress might offer some useful guidelines to develop positive psychology-based student counseling interventions to help Chinese students cope with adversity and hardship.

## 6. Limitations

Although we used a relatively large sample size and well-validated instrument scales, this study has some limitations. First, the data used in this study were obtained from a single source (i.e., university students). We reduced common method variance by using some negatively-worded items, ensuring anonymity and confidentiality. However, there is still a potential for common method variance to bias our study results. Future studies could adopt as many measures as possible, as recommended by Podsakoff et al. [29], to further minimize this issue. Second, this study was cross-sectional in nature, which precludes us from making a causal conclusion about the main and moderation effects. If possible, a longitudinal study or experimental design is recommended to interpret the relationships between incivility, academic stress, and psychological distress in a causal way. Third, despite the large sample size in this study, most participants came from the province where the university is located and no students from the three autonomous regions participated in this study. This sample distribution, together with a low response rate (4.58%), might bias the study results. Future studies could collect more data from other provinces in order to be more representative of the whole Chinese student population. Next, we only solicited data from one university in China. Although students were from all parts of China, the sample may not represent the whole college student population in China. Finally, we only used gratitude as a moderator to examine how incivility and academic stress impact students’ psychological health, ignoring other possible mediators and moderators. If more variables were examined in the study, such as rumination, personality traits, and students’ attribution, we could gain a higher understanding of the underlying mechanisms and boundary conditions about how, whether, and when such effects might be most likely to occur.

## 7. Conclusions

This study showed that both incivility and academic stress positively affect university students’ psychological health. It also demonstrated that gratitude moderates the relationship between incivility and psychological distress after controlling for age, gender, and family income. However, the interaction of gratitude and academic stress does not significantly impact university students’ psychological health. For highly grateful students, the relationship between incivility and psychological distress is stronger than those with low levels of gratitude. In addition, female students scored higher on gratitude than male students, whereas there were no differences between these two groups on perceived incivility, perceived academic pressure, and psychological distress. This study contributed insight into the moderating role of gratitude in the incivility–psychological distress relationship. To advance our understanding, future researchers could use more measures to combat common method bias, employ a longitudinal study or experimental design to create causal interpretation, and recruit more representative samples for the study results to be generalized.

Incivility is a rude or impolite attitude or behavior towards others. Considering the vagueness and prevalence of incivility, maintaining civility on campus still remains a great concern for most college administers [45]. Incivility interferes with a harmonious and cooperative learning atmosphere, contributing to increased psychological distress among college students. Given its widespread effect on both students and college culture, colleges and universities should take measures to tackle this disturbing issue. At the college or university level, administers should create a culture in which each and every student is treated with respect, fairness, and equality. At the department level, college counseling staff and department faculty should make it clear what behaviors students need to follow and what should be avoided. In addition, they should provide counseling to help change students’ behaviors. Furthermore, students as individuals also need to understand their own roles and assume corresponding responsibilities. They must stand up against any uncivilized behaviors occurring on campus in order to stop such behaviors. In this way, we can facilitate civility and enhance learning effectiveness among college students.

## Figures and Tables

**Table 1:** Means, standard deviations, reliabilities, and intercorrelations between variables.

Variable	M	SD	1	2	3	4	5	6	7
1. Age	2.14	1.12							
2. Gender	1.78	0.42	−0.06						
3. Income	2.34	1.27	0.03	−0.08 *					
4. Incivility	2.45	0.75	0.03	−0.06	−0.04				
5. Stress	2.98	0.56	0.02	0.04	−0.12 **	0.30 **			
6. Gratitude	3.89	0.63	−0.07 *	0.15 **	0.00	−0.10 **	−0.01		
7. Psychological distress	2.45	0.72	0.08 *	0.01	−0.04	0.37 **	0.52 **	−0.20 **	

Note: N = 895; M = mean; SD = standard deviation. * Correlation is significant at 0.05 level (2-tailed). ** Correlation is significant at 0.01 level (2-tailed).

**Table 2:** Model comparison.

Model	χ^2^	*df*	Δ χ^2^	RMSEA	CFI	TLI	SRMR
**Four-factor model** **IN, ST, GR, PS**	502.17	203		0.04	0.96	0.96	0.04
**Three-factor model** **IN + ST, GR, PS**	2289.18	206	1787.01 **	0.11	0.73	0.70	0.12
**Two-factor model** **IN + ST + GR, PS**	3793.65	208	3291.48 **	0.14	0.54	0.48	0.16
**One factor model** **IN + ST + GR + PS**	4759.14	209	4256.97 **	0.16	0.41	0.35	0.16

Note: N = incivility; ST = academic stress; GR = gratitude; PS = psychological distress. ** *p* < 0.01. Δ χ^2^ is χ^2^ difference between respective and four-factor models.

**Table 3:** Results of hierarchical multiple regression.

	Psychological Distress		
Variable	Model 1	Model 2	Model 3
**Control Variables**			
**Age**	0.09 ** (0.00)	0.06 * (0.04)	0.06 * (0.04)
**Gender**	0.01 (0.58)	0.04 (0.22)	0.04 (0.27)
**Income**	−0.05 (0.15)	0.02 (0.47)	0.02 (0.49)
**Predictors**			
**Incivility**		0.22 ** (0.00)	0.22 ** (0.00)
**Stress**		0.46 ** (0.00)	0.46 ** (0.00)
**Gratitude**		−0.18 ** (0.00)	−0.17 ** (0.00)
**Incivility × Gratitude**			0.07 * (0.02)
**Stress × Gratitude**			−0.05 (0.11)
***Δ R*^2^**	0.01 ** (0.00)	0.35 ** (0.00)	0.01 * (0.02)
**F**	2.82 * (0.03)	83.11 ** (0.00)	63.32 ** (0.00)

Note: ** *p* < 0.01; * *p* < 0.05. *p*-Values are in parentheses. All values were standardized regression coefficients.
